# miR-491 regulates glioma cells proliferation by targeting TRIM28 in vitro

**DOI:** 10.1186/s12883-016-0769-y

**Published:** 2016-12-01

**Authors:** Zengxin Qi, Shengyong Cai, Jiajun Cai, Lingchao Chen, Yu Yao, Liang Chen, Ying Mao

**Affiliations:** 1 Department of Neurosurgery, Huashan Hospital, Fudan University, 12 Wulumuqi Middle Road, Shanghai, 200040 China; 2 Department of Neurosurgery, Shanghai Medical College, Fudan University, Shanghai, China; 3 State Key Laboratory of Medical Neurobiology, School of Basic Medical Sciences and Institutes of Brain Science, Fudan University, Shanghai, China

**Keywords:** Glioblastoma, miR-491, TRIM28, Proliferation

## Abstract

**Background:**

MicroRNAs are significantly involved in tumorigenesis and progression of glioma. However, the critical part they play in glioma have not been fully elaborated. miR-491 and Tripartite motif containing 28 (TRIM28) are reported to aberrantly express in glioblastoma multiforme (GBM). Here, we detected miR-491 and TRIM28 expression and function in glioma cells.

**Methods:**

We analyzed miR-491 expressions in 20 primary human GBM tissues and 6 control brain tissues by qRT-PCR assays and searched for The Cancer Genome Atlas (TCGA) database. Then we predicted possible mRNA target of miR-491 by TargetScan/MicroRNA and confirmed it via luciferase reporter assays. Knock-down of miR-491 and transfection of pLenti-TRIM28 were performed in U251 and U87 cells. Proliferation ability was examined by MTT and clone formation assays.

**Results:**

miR-491 expression was obviously reduced in GBM cells and tissues. There was a positive correlation between the down-regulation of miR-491 and poor prognosis. Spearman’s correlation analysis demonstrated that miR-491 expression was negatively correlated with TRIM28 protein level. Possible mRNA binding sites of miR-491 predicted by TargetScan/MicroRNA were proved by luciferase assays. Clone formation and MTT assays indicated that up-regulation of miR-491 inhibited the proliferation of glioma cells.

**Conclusions:**

miR-491 regulates glioma cells proliferation in vitro by targeting TRIM28.

**Electronic supplementary material:**

The online version of this article (doi:10.1186/s12883-016-0769-y) contains supplementary material, which is available to authorized users.

## Background

Proteins of TRIM family belong to members of E3 ligases family, which play an important part in the process of cellular protein hydrolysis [1]. Until now, over 70 TRIM proteins were identified. Some of them were proved to be related to proliferation, apoptosis and transcriptional modulation of cancer cells [2]. TRIM28, a member of TRIM family, expression of which has been verified to be up-regulated in several tumors; for instance, lung cancer, breast cancer and gastric cancer [3, 4]. We demonstrated a positive correlation between TRIM28 expression and glioma malignancy in a previous study [5]. Both in vitro and in vivo, higher expression of TRIM28 lead to a worse prognosis via promotion of the proliferation ability of glioma cells.

MicroRNAs are small, non-coding RNAs which participate in modulating various kinds of biological processes [6, 7]. MiRNAs recognize their target sites by directly interacting with the 3’UTR of target mRNA. They could lead to degradation or translational repression of the target mRNAs [8]. What’s more, each miRNA could potentially modulate up to over one hundred mRNAs. It’s estimated that over one third of human genes are regulated by miRNAs [9]. Several literatures have elucidated the role which miR-491 plays in tumorigenesis and tumor progression. Nakano et al. demonstrated that miR-491 was up-regulated in colorectal cancer and induced apoptosis via the modulation of Bcl-XL [10]. Via inhibition of Epithelial-Mesenchymal Transition (EMT) and expression of matrix metalloproteinase, miR-491 is also related to metastasis of hepatocellular carcinoma [11].

Several studies indicated a correlation between the downregulation of miR-491 and tumor malignancy in GBM [12, 13]. X Li et al. [12] indicated that miR-491-5p could modulate the expression of Bcl-xL, CDK6 and EGFR, meanwhile CDK6 and IGFBP2 were the target of miR-491-3p. Bcl-xL, CDK6 and IGFBP2 are well-recognized carcinogenic gene. EGFR amplification is one of the most common gene changes happening in tumorigenesis of GBM. Therapeutic measures which target EGFR have shown promising outcomes in GBM patients [14, 15]. Moreover, MIR-491 is indicated always deleted together with CDKN2A, a tumor suppressor gene famous for encoding p16^INK4α^ and p14^ARF^. Interestingly, MIR-491’s host gene, KIAA1797/FOCAD, was well recognized to function as tumor suppressor gene of glioma. So the close relevance of spatial structure and regulatory relationship between MIR-491 and the widely recognized cancer related genes mentioned above indicated the tumor inhibition ability of miR-491 in GBM. Literature also demonstrated that TRIM28 was one of the 681 genes potentially targeted by miR-491 predicted by Targetscan, but without further verified [12]. Just because the important part miR-491 plays in gliomas and its possible regulation relationship with TRIM28, we conducted further researches.

In the present study, we investigated miR-491 expression and TRIM28 level in both our cohort and from The Cancer Genome Atlas (TCGA) database. The result demonstrate that miR-491 was low expressed in GBM tissues. miR-491 expression correlated negatively with TRIM28 protein levels. Down-regulation of miR-491 lead to enhanced expression of TRIM28 and promote the proliferation of glioma cells. We demonstrate that TRIM28 is a direct target of miR-491. This study indicates that miR-491 may serve as a potential diagnostic marker of GBM and restoration of miR-491 together with other combined treatments, may develop into an effective method for the treatment of gliomas.

## Methods

### Tissue specimens’ acquisition

We obtained 20 GBM tissues from patients diagnosed with GBM from January 2011 to December 2012 at Huashan Hospital. We got 6 control brain tissues from trauma patients or from epilepsy surgery in the corresponding period. All the patients signed informed consent. The Huashan hospital Institutional Review Board (HIRB) approve the acquisition of all tissues used.

### TCGA database and statistical analysis

We got expression data of mRNA and miRNA from the website below: http://tcga-data.nci.nih.gov/tcga/homepage.htm. The significance of differences between two groups was evaluated by *χ* ^2^ test and Student’s *t* test. Optimal cut-off for miR-491 expression based on survival analysis was obtained by using X-tile software version 3.6.1 (Yale University School of Medicine, New Haven, CT, USA) [16]. *P* < 0.05 were the standard for being statistically significant. The statistical software we used for statistical analysis was SPSS Graduate Pack 11.0 (SPSS, Chicago, IL, USA).

### Computational algorithm analysis

mRNA target sites of miR-491 was predicted through the algorithms TargetScan Human 5.1 (www.targetscan.org) and human microRNA (http://www.microrna.org/). We speculated the minimum free energy for hybridization and examined through BibiServ (http://bibiserv.techfak.uni-bielefeld.de/genefisher2/).

### Cell culture and transfection

We obtained HEK293T and glioma cell lines U251, U87, SHG44 from Cell Bank of Chinese Academy of Sciences, Shanghai, China. DMEM with 10% fetal bovine serum (GIBCO, Carlsbad, CA, USA) was used for cell culture. Cell transfections with plasmid vectors expressing TRIM28, pLenti-TRIM28 or miR-491 mimics (Genomeditech, Shanghai, China) were carried out with Lipofectamine 2000 (Invitrogen, Carlsbad, CA, USA).

### Colony formation assay and proliferation assay

Colony formation assay and proliferation assay were carried out as previously introduced [5]. We repeated every experiment at least three times.

### qRT-PCR assay

We performed total RNA isolation, cDNA synthesis and qRT-PCR as previously introduced [5]. GAPDH was used as an internal standard. Primers for TRIM28 were 5’-AGCGGGTGAAGTACACCAAG-3’ and 5’-ACCCAAAGCCATAGCCTTCC-3’, primers for GAPDH were 5’-TCCACCACCCTGTTGCTGTA-3’ and 5’-ACCACAGTCCATGCCATCAC-3’ (Genomeditch, Shanghai, China).

### Western blotting

Western blotting was carried out as previously introduced [5]. Rabbit anti-human TRIM28 (ab10483, Rabbit, 1:1000; Abcam, Cambridge, MA, USA) was selected to examine TRIM28 expression. β-actin (Abcam, MA, USA) was selected for an internal control. We repeated every experiment at least three times.

### Luciferase reporter assays

We synthesized the 3’UTR of TRIM28 containing the miR-491 target sequence (5’-UCCCCAC-3’) (Genomeditech, Shanghai, China) and inserted it into pGL3-control vector (Promega, Madison, WI), with resultant plasmids pGL3-TRIM28-wt. To determine sequence specificity, we also established the plasmids pGL3-TRIM28-mt in which the conserved targeting sequence of miR-491 was mutated to 5’-GUAAAGA-3’. HEK-293 cells were co-transfected with miR-491 mimics or scrambled miRNA and reporter plasmids using Lipofectamine 2000. Lucferase assys were detected by the dual-luciferase reporter assay system after 48 h (Promega, USA).

## Results

miR-491 is obviously down-regulated in glioma tissues and cell lines, which is correlated with poor prognosis.

**Fig. 1:**
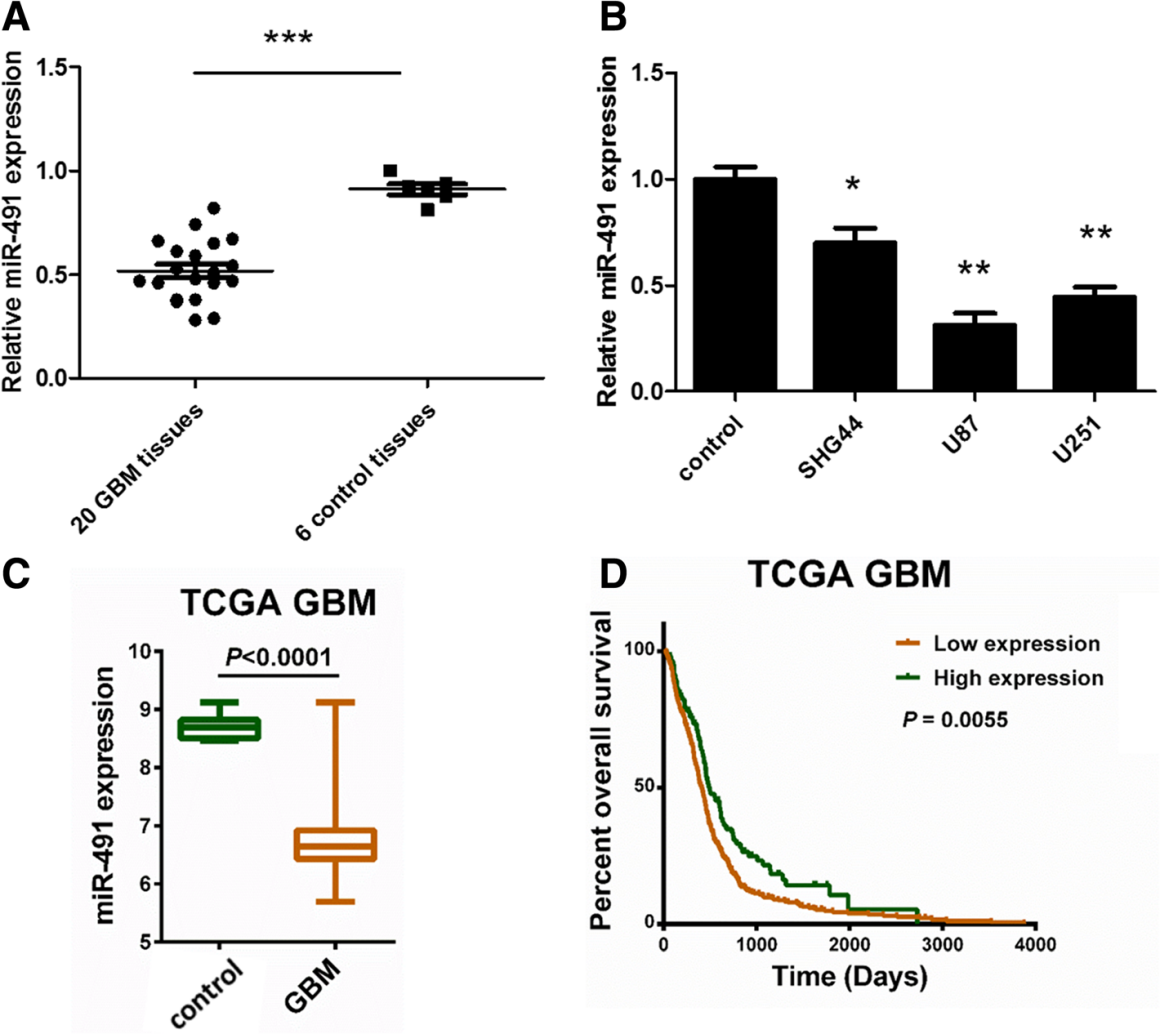
miR-491 is reduced in glioma, which is correlated with poor prognosis. **a** Expression of miR-491 in GBM tissues (*n* = 20) and control brain tissues (*n* = 6) tested by RT-qPCR assays. ***P < 0.001 **b** miR-491 expression is lower in SHG44, U87, U251 glioma cell lines than in control brain tissues. *P < 0.05, **P < 0.01. **c** Expression of miR-491 in GBM (*n* = 512) and control brain specimens (*n* = 10) from TCGA database. Data are shown as median and range. *P* < 0.0001. **d** Retrospective analysis of the overall survival from TCGA database demonstrated that decreased miR-491 expression represent poor prognosis in GBM. Cut-off =6.962409973. *P* = 0.0055

To investigate the role miR-491 plays in the tumorigenesis and progression in glioma, we analyzed miR-491 expressions in 20 primary human GBM tissues and 6 control brain tissues by RT-qPCR assays. It showed that miR-491 expressed lower in GBM tissues (***P < 0.001) (Fig. 1a). Next, we tested miR-491 expression levels in several glioma cell lines and got the similar results as shown in glioma tissues. miR-491 expressed lower in glioma cell lines SHG-44 (low grade), U87 and U251 (high grade) than normal brain specimens(*P < 0.05,**P < 0.01) (Fig. 1b). TCGA database also indicated a significant lower miR-491 expression in GBM (*n* = 512) than in control brain tissues (*n* = 10) (*P* < 0.0001) (Fig. 1c). What’s more, retrospective analysis of the clinical outcome from TCGA data demonstrated a positive correlation between decreased miR-491 and poor survival in GBM (*P* = 0.0055) (Fig.1d). 405 GBM cases were assigned to low expression group and the other 107 GBM cases were assigned to high expression group. (Additional file 1: Table S1) The cut-off value was 6.962409973 which was calculated by X-tile software. Above results may indicate that miR-491 may serve as tumor suppressor gene in glioma.

### TRIM28 is the target of miR-491

**Fig. 2:**
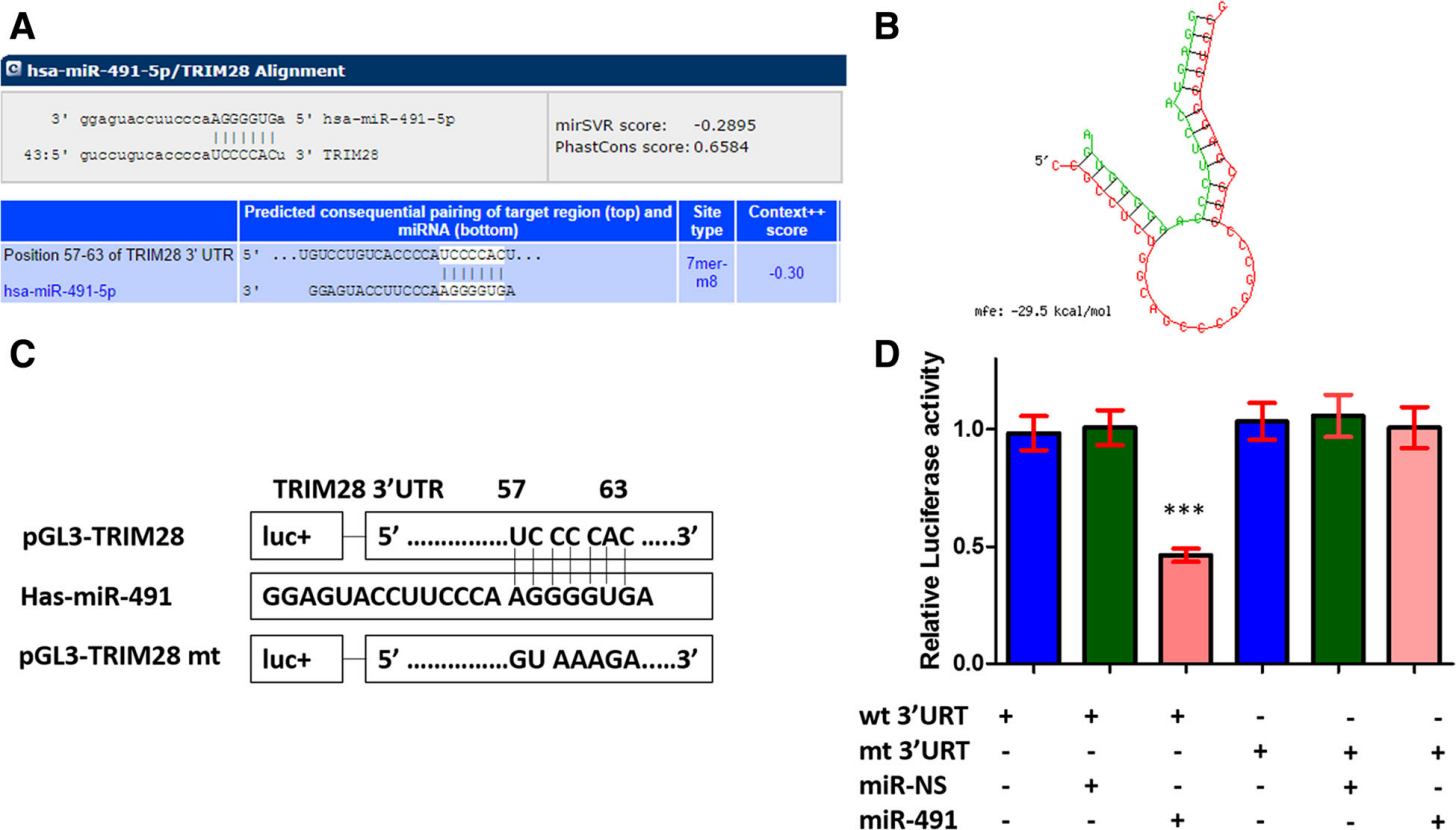
TRIM28 is directly targeted by miR-491. **a** The nucleotide 57-63 region within the 3’-UTR of TRIM28 are potential complementary site of miR-491. **b** BibiServ analysis of free energy (DG) for hybridization of miR-491 and 3’-UTR of TRIM28. **c** A schematic diagram of reporter which contained 3’UTR of TRIM28 was constructed. **d** Luciferase reporter assays were performed after co-transfecting with pGL3-TRIM28-wt or pGL3-TRIM28-mt alone, or together with miR-491 mimics or scrambled miRNA controls (NS). Data are listed as mean ± SD of three times. ***P < 0.001

Following searching for TargetScan and human microRNA database, we found the possible complementary base pair between seed region of miR-491 and nucleotide 57–63 region in the 3’-UTR of TRIM28 (Fig. 2a). BibiServ analysis showed that the free energy (DG) was approximately -29.5 kcal/mol for hybridization of the TRIM28 3’-UTR and miR-491 (Fig. 2b). Next, we synthesis the 3’UTR of TRIM28 containing miR-491 binding sites and a luciferase reporter gene, then inserted it into a pGL3 vector to confirm the direct interaction between miR-491 and the 3’UTR of TRIM28 (Fig. 2c). Luciferase reporter assays were performed following co-transfecting with pGL3-TRIM28-wt or pGL3-TRIM28-mt alone, or together with miR-491 mimics or scrambled miRNA controls (NS) (Fig. 2d). It showed that Luciferase activities in the group transfected with pGL3-TRIM28-wt and miR-491 mimics is significant lower than that in other groups (***P < 0.001). Above results indicated that TRIM28 is direct target of miR-491.

### miR-491 negatively regulates TRIM28 levels in GBM

**Fig. 3:**
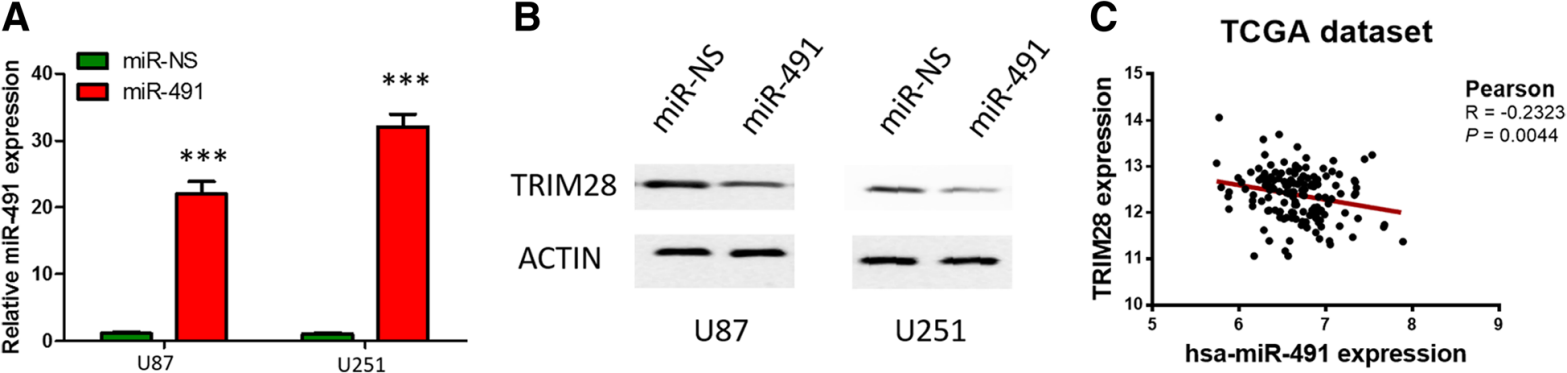
miR-491 negatively regulated TRIM28 levels in GBM. **a** U251 and U87 cells were transfected with miR-491 mimics or scrambled miRNA (miR-NS). miR-491 expression were examined by qRT-PCR assays. ***P < 0.001. **b** TRIM28 protein level was detected by western-blot assays. **c** Spearman’s correlation analysis of miR-491 expressions and TRIM28 protein levels (*r* = -0.2323, *P* = 0.0044)

In view of the tumor suppressor role miR-491 may play in glioma and miR-491 directly targeted the 3’-UTR of TRIM28, we correlated miR-491 expression with TRIM28 level. miR-491 mimics or scrambled miRNA controls were synthesized to knock down the expression of miR-491 and western-blot assay showed miR-491 mimics markedly suppressed TRIM28 expression at protein level (Fig. 3a and b). Next, we searched for TCGA data to investigate the relationship between expression of TRIM28 and miR-491 level. Spearman’s correlation analysis showed a negative correlation (*r* = -0.2323, *P* = 0.0044). Above findings suggested that TRIM28 was regulated in GBM tissues and cells by miR-491.

### miR-491 inhibits glioma cell proliferation by modulating TRIM28

**Fig. 4:**
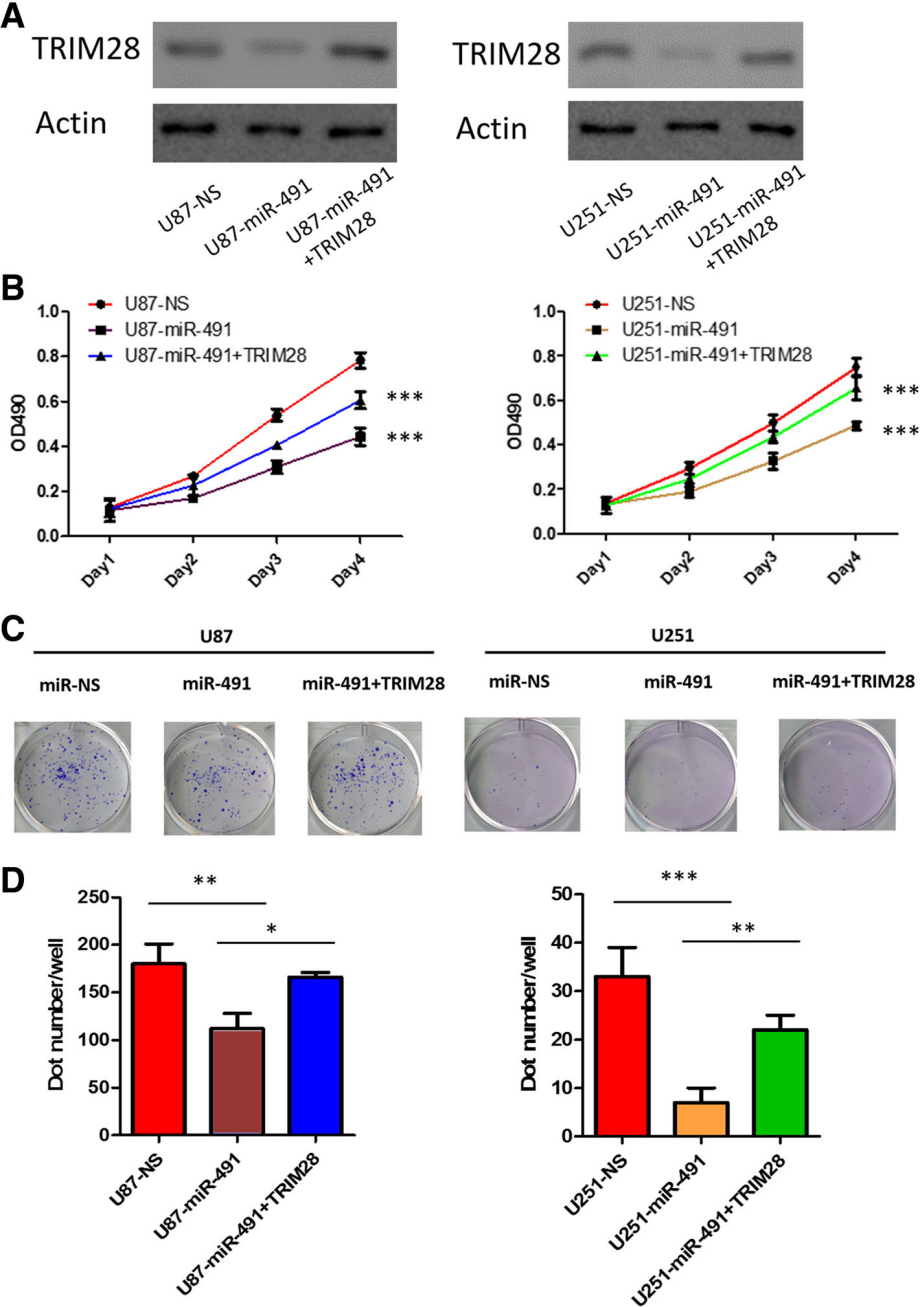
miR-491 inhibits glioma cell proliferation by modulating TRIM28. **a** U251 and U87 cells were transfected with scrambled microRNA (NC), miR-491 mimics or miR-491 mimics together with pLenti-TRIM28. TRIM28 protein expressions following transfection in U87 and U251 cells were tested by western-blot assay. **b** MTT assays were performed to detect cell proliferation ability. ***P < 0.001. **c** and (**d**) Clone formation ability was decreased following miR-491 transfection, which could be reversed by up-regulation of TRIM28 expression. Data are revealed in mean ± SD of in triplet. *P < 0.05, **P < 0.01, ***P < 0.001

miR-491 has been reported to suppress the proliferation of U251 cell lines. We wondered whether TRIM28 played any role in miR-491 mediated inhibition of glioma cell proliferation. Transfection of U87 and U251 cells with miR-491 mimics lead to up-regulated expression of miR-491 and an obvious reduction of TRIM28 protein level in both cell lines. However, the phenomenon was reversed by co-transfection with pLenti-TRIM28 (Fig. 4a). MTT assay (Fig. 4b) and clone formation (Fig. 4c) assay indicated that up-regulation of miR-491 decreased proliferation capacity of U251 and U87 glioma cells compared with NC-transfected. The suppression in the proliferation of U251 and U87 cells was significantly reversed by co-transfection with pLenti-TRIM28 (*P < 0.05, **P < 0.01, ***P < 0.001). These data indicated that miR-491 regulated the proliferation capability of gliomas cells via TRIM28.

## Discussion

Excessive proliferation of malignant gliomas is one of the main contributors to the poor prognosis of the disease. Further study of the physiological and molecular mechanisms of formation and progression of glioma is necessary to development more effective diagnostic and therapeutic approaches. Our previous study demonstrated that knockdown of TRIM28 in glioma cells suppressed cell proliferation, which could be partially explained by the negative correlation between TRIM28 and p21 expression in GBM patients [5]. Our present study indicated an aberrant expression of miR-491 in GBM specimens and that TRIM28 protein levels negatively correlates with miR-491 expression from TCGA database and our cohort. According to above results, we supposed miR-491 to serve as a tumor suppressor in glioma. In order to test the assumption, we constructed miR-491 transfected cell lines to performed functional analyses. As a result, up-regulation of miR-491 suppressed proliferation ability of glioma cells, which could be reversed by restoration of TRIM28. Then, we confirmed that TRIM28 was a direct target of miR-491 via a luciferase assay. TRIM28 protein was obviously up-regulated in U87 and U251 cells after transfection with miR-491, which also confirmed above conclusion.

Several studies indicated that miR-491-5p expression was reduced and acted as a potential cancer suppressor in various cancers, for instance, hepatocellular carcinoma, ovarian carcinoma, oral squamous cell carcinoma and pancreatic cancer [11, 17–19]. Deletion of MIR-491 has been recently reported to modulate the invasion and proliferation of GBM [12]. miR-491-5p and miR-491-3p are two mature miRNAs produced by MIR-491. Data from TCGA database support that expression levels of the two mature forms are correlated with each other. Loss of MIR-491 is negatively correlated with the overexpression of IGFBP2, CDK6, and EGFR. Loss of MIR-491 could regulate IGFBP2, CDK6 and EGFR proliferative pathways, by which the propagation of GBM cancer stem cells was inhibited. miR-491-5p were demonstrated to be negatively correlated with the expression of MMP-9 protein and reduced invasive ability of U251 and U87 glioma cell lines. 3’ UTR of MMP-9 was directly targeted by miR-491-5p, which was verified by luciferase assay [13]. As far as we know, this study confirmed that miR-491 directly targets TRIM28 in modulating proliferation activity of glioma cells in vitro for the first time.

TRIM28, a member of the TRIM family, was reported to promote or inhibit intracellular transcription according to the literature [20–22]. It was reported that TRIM28 acted as transcriptional corepressor via association with the histone deacetylase complex NuRD and the histone methyltransferase SETDB1, which resulted in the silencing of specific genes [23, 24]. TRIM28 can also lead to transcriptional activation via interaction with certain response elements, for example, the Nur response element (NuRE) [22]. TRIM28 was demonstrated to suppress p16 induction without affecting expression of p21 or p53 in lung fibroblast cell IMR90 [25]. In our previous study, we found the negative correlation of p21 and TRIM28 expression in glioma cells. P21 is a well-recognized cell cycle inhibitor and a tumor suppressor gene. Reduced p21 level plays an important part in tumorigenesis and progression. The negative correlation between p21 and TRIM28 indicated in our previous study could partly explain the role TRM28 played in the activation in proliferation of glioma cells. It is indicated that Sumoylated TRIM28 was related to the down-regulation of H3-K14 and H3-K9 acetylation. TRIM28 also took part in the acceleration of H3-K9 methylation at the p21 promoter [26]. Meanwhile, miR-491 may influence proliferation ability of glioma cells partly through p21 pathway.

## Conclusions

In conclusion, our present study indicates a common phenomenon that miR-491 is down-regulated in GBM and miR-491 is proved to be involved in modulating proliferation of glioma cells in vitro. miR-491 directly binds to 3’UTR of TRIM28 mRNA and suppresses TRIM28 expression. As a result, restoration of miR-491 together with other combined treatments may develop into an effective method for the treatment of gliomas.

## Additional file

Additional file 1: Table S1. miR-491 expression, OS and PFS of 512 GBM tissues from TCGA database. 405 GBM cases were assigned to low expression group and the other 107 GBM cases were assigned to high expression group. The cut-off value was 6.962409973 which was calculated by X-tile software. (XLSX 29 kb)

## Abbreviations

EMT: Epithelial-Mesenchymal Transition
GBM: Glioblastoma multiforme
qRT-PCR: Quantitative real-time polymerase chain reaction
TCGA: The Cancer Genome Atlas
TRIM28: Tripartite motif containing 28
